# Supplementary material for: Empirical methods for controlling false positives and estimating confidence in ChIP-Seq peaks
Source: BMC Bioinformatics. 2008 Dec 5;9:523. doi: 10.1186/1471-2105-9-523 (PMC2628906; doi:10.1186/1471-2105-9-523)
Supplement: Additional File 1 — A variety of html documents from the USeq web site detailing the available applications, their best usage, output file type descriptions, command line menus, etc. [file 1471-2105-9-523-S1.zip › USeqUserGuides/JavaDoc.zip!/JavaDoc/gata/main/GATAPlotter.html]

GATAPlotter


|  |  |  |
| --- | --- | --- |
| **Overview** \| **Package** \| **Class** \| **Use** \| **Tree** \| **Deprecated** \| **Index** \| **Help** | |  |
| **PREV CLASS**   **NEXT CLASS** | **FRAMES**    **NO FRAMES**     **All Classes** |  |
| SUMMARY: NESTED \| FIELD \| CONSTR \| METHOD | DETAIL: FIELD \| CONSTR \| METHOD |  |


---


## gata.main Class GATAPlotter

```
java.lang.Object
  gata.main.GATAPlotter
```

---

``` public class GATAPlotter extends java.lang.Object ```

**Author:**
:   Nix
    Launcher class for GATAPlotter. Call main.

---

**Constructor Summary**

`GATAPlotter()`


| **Method Summary** | |
| --- | --- |
| `javax.swing.JScrollPane` | `getAlignScrollPane()` |
| `javax.swing.JScrollPane` | `getCompScrollPane()` |
| `javax.swing.JSplitPane` | `getInnerSplitPane()` |
| `java.io.File` | `getObjectsFile()` |
| `void` | `launchGATAPlotter()` |
| `static void` | `main(java.lang.String[] args)` |
| `void` | `makeAnnotation(AnnoSpecParams annoParams)` |

**Methods inherited from class java.lang.Object**

`equals, getClass, hashCode, notify, notifyAll, toString, wait, wait, wait`

**Constructor Detail**

### GATAPlotter

```
public GATAPlotter()
```


**Method Detail**

### main

```
public static void main(java.lang.String[] args)
```

---


### getObjectsFile

```
public java.io.File getObjectsFile()
```

---


### launchGATAPlotter

```
public void launchGATAPlotter()
```

---


### makeAnnotation

```
public void makeAnnotation(AnnoSpecParams annoParams)
```

---


### getCompScrollPane

```
public javax.swing.JScrollPane getCompScrollPane()
```

---


### getAlignScrollPane

```
public javax.swing.JScrollPane getAlignScrollPane()
```

---


### getInnerSplitPane

```
public javax.swing.JSplitPane getInnerSplitPane()
```


---


|  |  |  |
| --- | --- | --- |
| **Overview** \| **Package** \| **Class** \| **Use** \| **Tree** \| **Deprecated** \| **Index** \| **Help** | |  |
| **PREV CLASS**   **NEXT CLASS** | **FRAMES**    **NO FRAMES**     **All Classes** |  |
| SUMMARY: NESTED \| FIELD \| CONSTR \| METHOD | DETAIL: FIELD \| CONSTR \| METHOD |  |


---
